# ART adherence clubs in the Western Cape of South Africa: what does the sustainability framework tell us? A scoping literature review

**DOI:** 10.1002/jia2.25235

**Published:** 2019-03-19

**Authors:** Kornelia Flämig, Tom Decroo, Bart van den Borne, Remco van de Pas

**Affiliations:** ^1^ Maastricht Centre for Global Health Maastricht University Maastricht The Netherlands; ^2^ Department of Clinical Sciences Institute of Tropical Medicine Antwerp Belgium; ^3^ Research Foundation Flanders Brussels Belgium; ^4^ Faculty of Health, Medicine and Life Science Maastricht The Netherlands; ^5^ Department of Public Health Institute of Tropical Medicine Antwerp Belgium

**Keywords:** antiretroviral therapy, implementation process, patient participation, sustainability, adherence club, review, ART delivery model, South Africa

## Abstract

**Introduction:**

In 2007, the antiretroviral therapy (ART) adherence club (AC) model was introduced to South Africa to combat some of the health system barriers to ART delivery, such as staff constraints and increasing patient load causing clinic congestion. It aimed to absorb the growing number of stable patients on treatment, ensure quality of care and reduce the workload on healthcare workers. A pilot project of ACs showed improved outcomes for club members with increased retention in care, reduced loss to follow‐up and a reduction in viral rebound. In 2011, clubs were rolled out across the Cape Metro District with promising clinical outcomes. This review investigates factors that enable or jeopardize sustainability of the adherence club model in the Western Cape of South Africa.

**Methods:**

A scoping literature review was carried out. Electronic databases, organizations involved in ACs and reference lists of relevant articles were searched. Findings were analysed using a sustainability framework of five key components: (1) Design and implementation processes, (2) Organizational capacity, (3) Community embeddedness, (4) Enabling environment and (5) Context.

**Results and Discussion:**

The literature search identified 466 articles, of which six were included in the core review. Enablers of sustainability included the collaborative implementation process with collective learning sessions, the programme's flexibility, the high acceptability, patient participation and political support (to some extent). Jeopardizing factors revolved around financial constraints as non‐governmental organizations are the main supporters of ACs by providing staff and technical support.

**Conclusions:**

The results showed convincing factors that enable sustainability of ACs in the long term and identified areas for future research. Community embeddedness of clubs with empowerment and participation of patients, is a strong enabler to the sustainability of the model. Further policies are recommended to regulate the role of lay healthcare workers, ensure the reliability of the drug supply and the funding of club activities.

**Abbreviations**
AC: Adherence club
ART: Antiretroviral therapy
ARV: Antiretroviral
CAC: Community adherence club
CAG: Community ART group
CDU: Central dispensing unit
DC: Differentiated Care
DoH: Department of Health
IAS: International AIDS Society
LHCW: Lay healthcare worker
MSF: Médecins Sans Frontières
NGO: Non‐governmental organization
NPO: Non‐profit organization
PLHIV: People living with HIV
RIC: Retention in care
SA: South Africa
TB: Tuberculosis
UNAIDS: United Nations Programme on HIV/AIDS
WHO: World Health Organizsation

## 1 Introduction

In 2016, South Africa (SA) reached the 7 million threshold of people living with HIV (PLHIV) 1 while providing the largest antiretroviral therapy (ART) programme worldwide, serving approximately 3.7 million people with treatment 2. To end the AIDS epidemic, the Joint United Nations programme on HIV/AIDS (UNAIDS) set the ambitious global 90‐90‐90 target in 2014 3. By 2020, 90% of PLHIV should know their status, at least 90% of HIV‐infected people should have access to ART and at least 90% of all people on ART should have viral suppression 3. Additionally, updates from the World Health Organization (WHO) on ART guidelines recommend the initiation of ART immediately after HIV diagnosis 4. Current data of PLHIV in SA show that 86% know their status, 56% are on treatment and only 45% are virally suppressed 1.

However, understaffed health systems with weakened infrastructure are increasingly under pressure to achieve the 90‐90‐90 goals 5. Factors include congestion in clinics with longer waiting hours and transportation costs to collect medicines. Competing demands on individuals of work, family and social life and time spent queuing in an overcrowded health centre, compounded by disappointment in quality of care, all have a negative impact on long‐term retention in care (RIC) and long‐term adherence 6, 7, 8.

**Figure 1:**
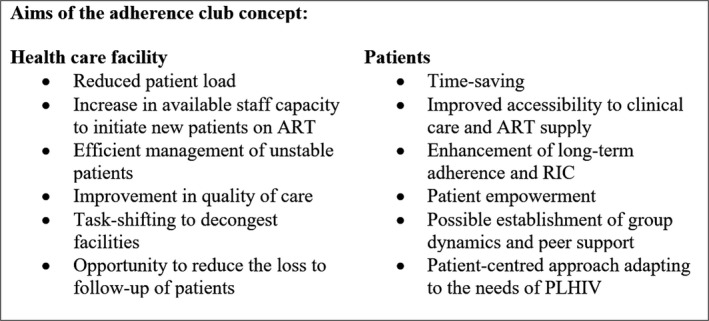
The aims of the club concept for patients and providers ART, antiretroviral therapy; RIC, retention in care; PLHIV, people living with HIV.

**Figure 2:**
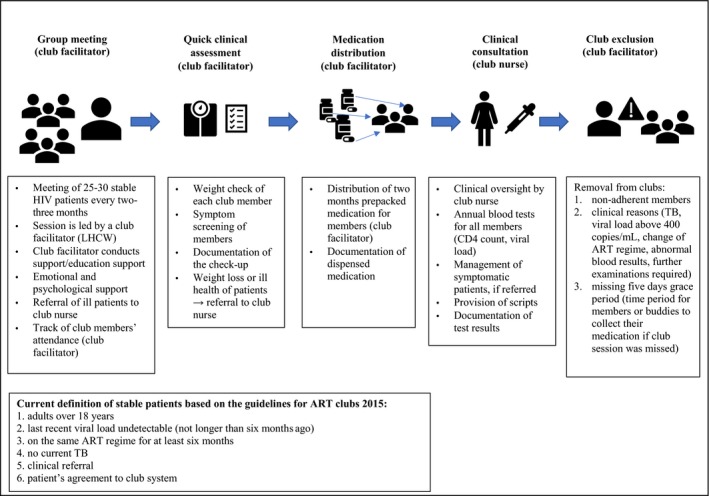
An illustration of activities during a club session Adopted from: Mukumbang *et al*.^13^. ART, antiretroviral therapy; LHCW, lay healthcare worker; TB, tuberculosis.

To combat these challenges ART adherence clubs (ACs) were introduced as a pilot project of Médecins Sans Frontières (MSF) at the Ubuntu Clinic, Khayelitsha, in Cape Town in 2007 9. In practice, ACs are groups managed by lay healthcare workers (LHCWs) for stable HIV patients on ART, which are separated from clinical visits and partially located in communities. In ACs, patients have a quick clinical assessment, receive support for adherence and get a refill of HIV medication 8, 9. Details of club activities and their aims have been published 8, 10, 11, 12, 13. Figure 1 and 2 summarize these.

ACs present a promising intervention, addressing poor long‐term RIC and suboptimal adherence, using a multipronged adherence‐enhancing approach such as patient education and peer support, also recommended by the WHO 14. According to the analysis of MacGregor *et al*. 15, an estimated number of 42.600 patients (32%) of 142.000 ART patients were enrolled in ACs throughout the Cape Metro Health District in March 2016. Due to improved outcomes of Cape Town's ACs, which led to lower loss to follow‐up and high levels of viral suppression and RIC 9, 10, 16, district authorities plan to accommodate about 70% to 90% of all clinically stable patients in ACs 15.

The South African national strategy for HIV, tuberculosis (TB) and sexually transmitted infection has several objectives; (1) initiation of ART for at least 80% of eligible patients, (2) achievement of the 90‐90‐90 goals by 2020, (3) ending AIDS and TB as major health threat by 2030 and (4) reaching out to key and vulnerable groups 2, 17.

The rollout of the AC model in SA 7, could be seen as part of this national effort on HIV, therefore, it is of interest to analyse the sustainability of early experiences with ACs. We conducted a scoping review to identify factors that enable or jeopardize the sustainability of the AC model.

## 2 Methods

**Figure 3:**
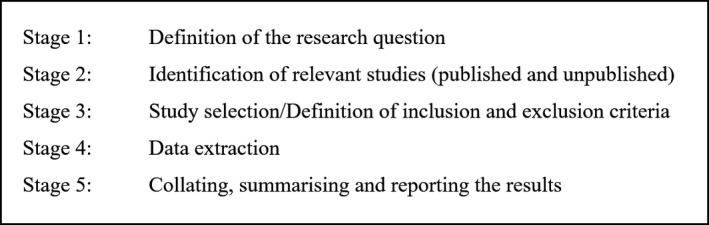
Five stage approach of a scoping literature review based on Arksey *et al*. 18

A scoping literature review was carried out, following a five‐step approach laid out by Arksey *et al*. 18 (Figure 3).

### 2.1 The framework of sustainability of adherence clubs

**Figure 4:**
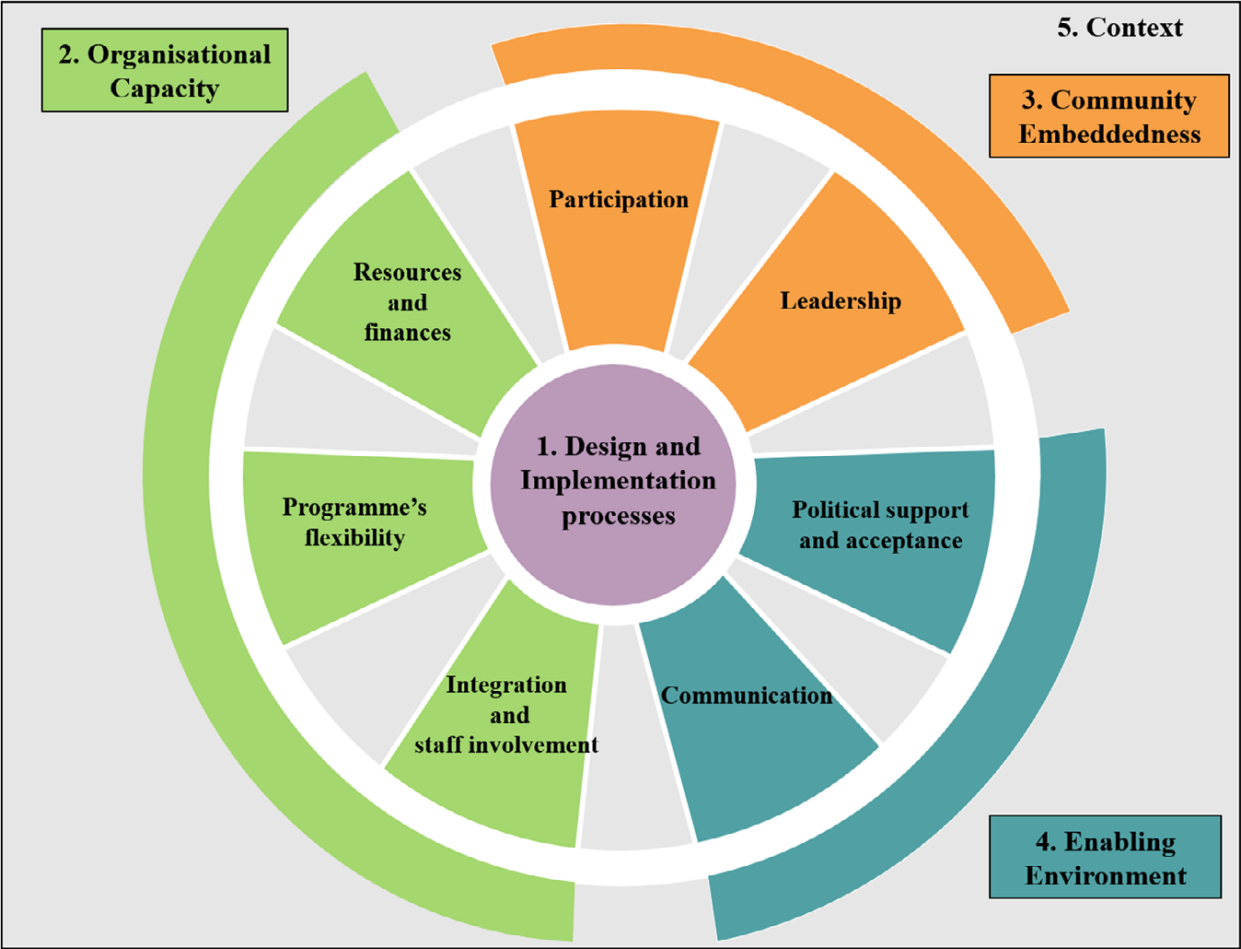
The conceptual framework on sustainability of ACs Adopted from: Rasschaert *et al*. 19.

The analysis on sustainability of ACs followed the framework of Rasschaert *et al*. 19 which is based upon the findings of Schell *et al*. 20 and Sarriot *et al*. 21. The framework of sustainability constitutes five key components: (1) *design and implementation processes*, (2) *organizational capacity*, (3) *community embeddedness*, (4) *enabling environment* and (5) *context* (Figure 4) 19. Table 1 presents a definition of each component of the framework.

**Table 1:** Definition of each component which constitutes the sustainability framework

Components	Definition
Design and implementation processes	Approaches and activities that are introduced to accomplish effectively the objectives and goals of a project. Elements such as building capacity, training, project duration, project effectiveness, project type and the process of negotiation with other stakeholders underpin the design and implementation process 19, 22. Project leaders are required for project support and negotiation 22
Organizational capacity	The ability of the model to function independently and to comply with the essential activities considering resources, finances and the ability to adapt to beneficiaries’ needs 21. The integration of an intervention into a system or institution is seen as crucial since it may serve as a supportive structure to sustain the initial programme 22
Community embeddedness	The achievement of community competence including social collectiveness, leadership and cohesion through community participation and leadership 21
Enabling environment	Political support 20 and national/regional policies contributing to the acceptance of the project 21. The political environment will influence the programme outcome and its sustainability 22
Context	All four components are embedded in the larger context including economic, political, cultural, environmental and geographical factors 19. These factors cannot be controlled directly 19. Ignoring local context due to rush for success may not be in favour for long‐term benefits 23

### 2.2 Literature search strategy and eligibility criteria

The search strategy involved three separate activities: (1) electronic database searching, (2) a web search and (3) snowballing of citations from reference lists of other authors. For part one, the following electronic databases were searched: EMBASE, MEDLINE, PubMed, and Web of Science. For the databases of MEDLINE, EMBASE and Web of Science, the first 100 listed results of each search were taken into account.

Secondly, a web search was undertaken including websites from non‐governmental organizations (NGOs) such as MSF and Kheth'Impilo and the Treatment Action Campaign. In addition, websites of organizations and institutions were assessed: the International AIDS Society (IAS), the IAS conference website and Differentiated Care (DC) website, the South African National AIDS Council, the South African Health News Service, the WHO, the Global Fund, UNAIDS, the South African Medical Research Council and the provincial and national Department of Health (DoH) of SA.

As third search strategy, a snowball technique was applied by reviewing the bibliography of relevant articles and related articles.

The following search string was used: (“adherence club” OR “antiretroviral treatment adherence club” OR “chronic ARV club” OR “community adherence club” OR “community adherence group” OR “differentiated care model” OR “community‐based antiretroviral care delivery”) AND “South Africa” AND “HIV”.

Table 2 shows the time frame, study settings, study designs, language and population criteria used for study inclusion.

**Table 2:** The defined criteria for study inclusion

Criteria	Definition
Time frame	2007 (piloting ACs) to July 2017 (end of the 9^th^ IAS Conference on HIV in Paris)
Study setting	South Africa
Study design	Peer reviewed (qualitative, quantitative and mixed‐method) and non‐peer reviewed articles
Language	English
Exposure	ACs and community ACs
Study population	All HIV patients receiving ART who are members of ACs, regardless of age and gender
Grey Literature (non‐peer reviewed)	Unpublished papers that showed original data with a method and a result section

AC, adherence clubs; ART, antiretroviral therapy; IAS, International AIDS Society.

To be included, articles had to show data on at least one component of the theoretical framework shown in Figure 4. All articles that investigated other interventions, such as home‐based care models or fast track distribution models, were excluded. Records that focused on other chronic disease clubs, other than HIV, and quantitative studies that investigated only clinical outcomes of AC, such as adherence, RIC and loss to follow‐up were not included in the review. Non‐English papers, studies outside of SA and media reports were disregarded. In addition, published and unpublished grey literature, including reports and guidelines, without original data or those not specifying the methods used for their findings were excluded.

### 2.3 Data collection

All literature identified based on the search strategies were imported into the reference management software, Mendeley Desktop (version 1.17.11) for screening and managing records, as well as to remove duplicate references.

The selection of all identified studies was conducted in accordance to a four‐step process: (1) title screening, (2) abstract scanning, (3) applying inclusion and exclusion criteria and (4) reading the complete article. If screening did not exclude records based on title and abstract, full text versions were assessed. In the end, full text versions of all potentially relevant articles were obtained.

### 2.4 Data extraction

Data of all selected records were extracted and recorded in an Excel spreadsheet using the following categories: (1) study citation and location, (2) study purpose, (3) study design and methods, (4) components and elements found in records based on the sustainability framework (*design and implementation, integration and staff involvement, programme's flexibility, resources and finances, participation, leadership, communication, political support and acceptance* and *context*), (5) limitations and (6) conclusions/recommendations (Appendix S1).

### 2.5 Analysis

A thematic analysis method 24 was used for identifying, analysing and reporting the framework components in the retrieved records. All citations were screened, carefully examined and interpreted through the lens of the framework. Data on components and elements of the framework were thematically organized and stratified by their enabling or jeopardizing effect on the sustainability of the AC model (Appendix S1).

## 3 Results

**Figure 5:**
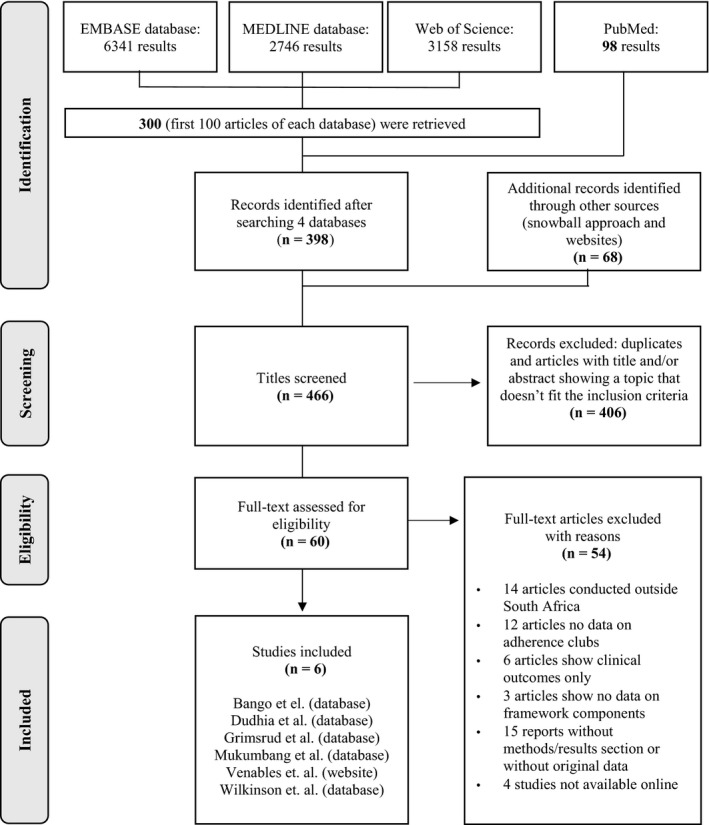
PRISMA Flow chart displaying the different phases of the review

Applying the key search term yielded 98 records in total from the electronic database of PubMed and all of them were screened. The electronic database search was complemented by the databases of EMBASE, MEDLINE and Web of science and resulted in 6341 records, 2746 records and 3158 records respectively. From each source the first 100 titles were screened. This approach retrieved citations with relevant titles including the publication that described the AC model for the first time. Another 68 records were retrieved from other sources including websites and snowball sampling. In total, 466 records were used for the identification process, of which 406 were excluded after reading the title and/or abstract and removing duplicates. 60 records were selected for the full‐text review and ultimately six records were retained for the core review. In Figure 5 the screening process is outlined in a PRISMA flow chart, and the characteristics of the included records are presented in Table 3. Of these six records, three are qualitative studies, with one poster presentation 25, 26, 27, one is a mixed‐method studies 6, while two are quantitative studies 28, 29.

**Table 3:** Characteristics of included records

Study citation and location	Type of document	Objective of study		Study design	Special Remarks		
					Factors enabling sustainability	Factors jeopardizing sustainability	
Bango *et al*. (2016). 6 Adherence clubs for long‐term provision of antiretroviral therapy: cost‐effectiveness and access analysis from Khayelitsha, South Africa. South Africa	Peer‐reviewed article	Assessment of the cost‐effectiveness of clubs in comparison to conventional care and analysis of the accessibility of club models		Mixed method (retrospective longitudinal study and interviews of club members)	Recognition that ACs are effective due to evidence for cost‐effectiveness Affordability and acceptability high for clubs	Persistence of stigmatization	
Dudhia *et al*. (2015). 25 Experiences of participating in an antiretroviral treatment adherence club. South Africa	Peer‐reviewed article	Analysis of the experiences of club participants and healthcare workers		Qualitative (interviews with members, doctors, counsellor. pharmacists)	Clubs encourage leadership (patient empowerment) Participation in patients’ treatment (peer support)	Challenge of club integration (linkage of CDU system/pharmacy/clinic shows problems) Identifies the need for sufficient resources (reliable drug supply)	
Grimsrud *et al*. (2015). 28 Implementation of community‐based adherence clubs for stable antiretroviral therapy patients in Cape Town, South Africa. South Africa	Peer‐reviewed article	Description of implementation of community adherence clubs and analysis of early clinical outcomes		Retrospective cohort study	Recognition of clubs’ effectiveness (high patient uptake, clinical outcomes) Recognition that clubs are extensions of facilities (club integration)	Identifies the challenge of insufficient resources (maintenance of community venues) Dependence on NGOs for staff and technical support Potential risk to poor linkage between clinics and clubs	
Mukumbang *et al*. (2016). 26 Towards developing an initial programme theory: Programme designers and managers assumptions on the antiretroviral treatment adherence club programme in primary healthcare facilities in the metropolitan area of Western Cape Province, South Africa. South Africa	Peer‐reviewed article	Evaluation of the adherence club programme based on the realist approach (to answer questions and identify what is functioning for whom, under which circumstances)		Qualitative (in‐depth interviews with designers and implementers) and review of documents	Strong support for club concept from all stakeholders favouring implementation process Recognition of leadership role of steering committee for successful implementation Patient participation (peer support)	Identifies the importance of government support through policies Dependence on NGO Identifies the need for resources (venue for meetings and its maintenance)	
Venables *et al*. (2017). 27 “If I'm not in the club, I have to move from one chair to another.” A qualitative evaluation of patient experiences of adherence clubs in Khayelitsha and Gugulethu, South Africa. South Africa.	Conference Presentation	Analysis of perceptions of clubs including club members and non‐members		Qualitative (focus group discussion and in‐depth interviews)	Recognition of club effectiveness (time‐saving, peer support) High acceptance among HIV patients	Lacking trusting relationship (patient‐clubs‐facilities) Identifies the need for adequate club integration to guarantee a functioning referral system between clubs and facilities	
Wilkinson *et al*. (2016). 29 Expansion of the adherence club model for stable antiretroviral therapy patients in the Cape Metro, South Africa 2011‐2015. South Africa	Peer‐reviewed article		Description of the scaling‐up process of adherence clubs across the Cape Metro district	Longitudinal cohort study	Recognition of role of steering committee in scaling‐up process Increasing number of patients and clubs (high acceptance) Clubs flexibility (eligibility criteria, different club models etc.)	Identification of financial resources (funding) for further scale‐up Identification of the need for sufficient human resources

AC, adherence clubs; CDU, central dispensing unit; LHCWs, lay healthcare workers; NGO, non‐governmental organization.

A summary of elements found to be either enabling or jeopardizing the sustainability of ACs are presented in Table 4.

**Table 4:** Summary of the main factors identified enabling or jeopardizing the sustainability of ACs

Components to sustainability	Enabling factors	Jeopardizing factors
Design and implementation process	Steering committee as leader and supporter (collaborative approach) Learning sessions/Feedback loop	Not identified in the core literature review
Organizational capacity	Linkage of clubs and clinic (well‐functioning club integration) Programme flexibility (adjusting eligibility criteria, extension of drug supply, introduction of specialized clubs)	Poor referral system between clinic and clubs (club integration) Insufficient resources and finances (dependence on NGOs, unreliable drug supply, staff capacity, space)
Community embeddedness	Social support/Peer support (patient participation) Leadership (patient empowerment)	Stigma
Enabling environment	Political support in rollout process High acceptance among all stakeholders	Lack of policies concerning recognition of LHCWs, drug supply Lack of standard operating procedures for example, for specialized clubs
Context	Recognition of local context	Not mentioned in the literature

AC, adherence club, LHCWs, lay healthcare workers, NGOs, non‐governmental organizations.

### 3.1 Design and implementation processes

The rollout of ACs across the Cape Metro District in 2011 demonstrated great success in organization and implementation 26. Firstly, this achievement was based on a collaborative approach between the Western Cape Provincial DoH, City of Cape Town Health Department, MSF, and the Institute for Healthcare Improvement to support the implementation with a so‐called steering committee 26. Secondly, a strategy of quality improvement was established, expanding the club model based on a collaborative short‐term learning system 26. In practice, mentors and the steering committee provided learning sessions for the club teams to guide implementation and make use of feedback loops to report back on progress and any emerging challenges 26. The steering committee undertook a key role in implementation and development of ACs 26. The literature highlighted the dependence on NGOs and non‐profit organizations (NPOs) for providing training and mentoring for club staff 26, 28. However, the review provided no estimates of required LHCWs in the light of increasing numbers of ACs and did not suggest strategies on organizational and financial sustainability while expanding staff capacity.

### 3.2 Organizational capacity

*Staff involvement* and *integration* are crucial factors to the functioning of the clubs 28. However, little is known about how this was successfully translated to the club setting. These two factors are important for the referral of patients to the club nurse to enable immediate clinical assessment and ensure high quality of care for the patient. The term *staff involvement* refers to the participation and communication of the whole team including health facility staff and club staff to secure the successful treatment of patients outside clinics 28. *Club integration* is linking clubs and facilities, to keep patients under the responsibility of the facilities, which remain accountable for club patients 28. One study indicated communication challenges between clubs and clinics 28.

The second element, *programme flexibility* of ACs indicates the ability to modify club features. These adjustments include changing eligibility criteria to enable participation in ACs. For example, only one undetectable recent viral load is required as opposed to previously requiring two 29. Further adjustments include being on treatment for only six months (instead of 18 months), receiving first‐ or second‐line ART and removing the CD4 count criterion 29. Community adherence clubs (CACs) were introduced where members meet at patients’ home or venues close to the community 28. In this way the club model became more flexible to meet patients’ interests and enable full decentralization of clubs 28.

*Resources and finances* are necessary for clubs to operate efficiently 29. Yet, findings of the review pointed out that information on the current financial situation for the clubs is incomplete. So far, the only study on cost‐effectiveness of ACs provided evidence on reduced costs of club‐provided care in comparison with a conventional healthcare service 6. The large contribution from NPOs and NGOs were seen as critical to the long‐term existence of the clubs 26. The utilization of the central dispensing unit (CDU) system was introduced to combat the pressured workload on pharmacy staff and to reduce waiting times 29. The outsourced medication distribution has potential pitfalls. Communication and logistical problems between pharmacies and the CDU such as lost scripts and late delivery of CDU pre‐packed medicine impaired the club operation 25.

### 3.3 Community embeddedness

The clubs facilitate *participation* of patients and create patient leaders 25. Studies showed that regular meetings led to peer support and the formation of social bonds among club members 25, 26. Sharing challenges and information provide mutual support in coping with HIV 26. This also leads to greater awareness about healthy living and helps communities to become healthier 26. In addition, stigma around HIV was reduced through regular group meetings 26 but not completely eradicated as some patients prefer to remain anonymous to their community 25. Also, patient *leadership* allows patients to become self‐determined and take responsibility for their own treatment 25, 28.

### 3.4 Enabling environment

*Communication* strategies of the AC model keep patients informed about alternatives to conventional care for example, waiting room talks by peer educators 27. The review did not show results from qualitative studies on effectiveness of communication on club participation.

The Western Cape government has provided *political support* and guidance to the rollout process in the Cape Metro District 26.

The study by Wilkinson *et al*. 29 showed high *acceptance* of ACs as numbers of club patients rose from 5683 to 32.425 with the number of clubs going up from 239 to 1308 between 2011 and 2015. A similar trend was found among the CACs in Gugulethu which had 74 clubs by 2013 28. Both providers and users reported experiencing the club meetings as positive and enjoyable 25.

### 3.5 Context

The retrieved literature did not provide much evidence on how current conditions in SA influence the function and development of ACs. Local economic, political and socio‐economic conditions and their impact on ACs to function optimally were neglected. Also, records gave no insight whether certain provinces in SA support the implementation process compared to others. According to the study of Grimsrud *et al*. 28, the introduction of the club model in Gugulethu benefited from well‐functioning logistics such as a good pharmacy system, as well as substantial NGO support. Therefore, it is unknown to which extent this club model is transferable to other settings.

## 4 Discussion

This review analysed the sustainability of ACs in the Western Cape based on the sustainability framework and identified factors enabling or jeopardizing the sustainability of the AC model. In total, six records documented the sustainability of ACs in the Western Cape. The first 100 titles of articles in each database were reviewed. All relevant citations were highly likely to be identified through this approach for several reasons: (1) all 98 citations of PubMed were ranked according to the publication date and all of them were screened, (2) retrieved citations included the first publication on ACs, (3) PubMed database included the latest citation on ACs, (4) PubMed citations were complemented by the most 100 relevant records retrieved from three other electronic databases, (4) use of snowball sampling and website search for literature identification and (5) all electronic databases showed repeatedly the same publications about ACs. These factors support the argument for completeness of our results.

The key factors enabling sustainability of the clubs are: (1) collaborative process to implement ACs, (2) flexibility to adjust to patient needs, (3) patient participation, (4) political support and (5) overall acceptance of the model. Among the main risks to the model's sustainability are resources and finances. Currently, funding for training, venues and technical support is mainly supplied by NGOs. CACs are largely managed by NGOs and the club counsellor is often employed by NGOs. As a result, there is uncertainty as to who will provide the long‐term running costs of the clubs. Funding is required for additional staff, staff training, space for club meetings and its maintenance and transportation of material 28, 29. Other jeopardizing factors for sustainability are potentially poor communication between clubs and clinics and a lack of policies and guidelines.

### 4.1 Findings of excluded literature

Another report, not retrieved through the search strategy, pointed out that the key role of the steering committee continues to be vital for the overall management of the clubs given the great demand for them 15. As the success of the clubs increase, the management and operation of clubs becomes more complex, which also affects the role of the steering committee. As a result, the information exchange between piloting facilities and steering committee is impaired due to increased workload 15. Other sources that have not been included in the review confirmed our results. Wilkinson 7 agreed that the successful implementation process is based on a collaborative approach with collective learning sessions. Other benefits included the opportunity for doctors to focus on patients who require more intensive care 30. However, poor bonds between clubs and clinics may affect clinical outcomes. One study revealed the lack of trusting relationships, poor communication between clubs and clinics and fear of decreasing quality in care due to task‐shifting of roles of the staff 15. Several NGO reports underlined the importance of community participation both in their therapy and implementation process 31, 32, 33, 34. The programme's flexibility was also recommended by various organizations with the emphasis to move away from the “one size‐fits‐all” approach 34, 35, 36, 37. To develop an adequate model, NGO reports highlighted the importance of local conditions such as health service capacity, availability of antiretrovirals (ARVs) and its regulation 31, 32, 33. Political support led to the government's endorsement of clubs to support linkage, adherence and RIC. The club concept was integrated in the National Adherence Guidelines for HIV, TB and non‐communicable diseases 37. Further assistance from the government was ensured through club guidelines. The guideline determines the roles of the club team members and provides information on eligibility criteria, the promotion of clubs, data collection and reporting, features of a club session and the response to a missed appointment 15. The provincial government of KwaZulu‐Natal also collaborated with MSF to rollout ACs and community ART groups (CAGs) across this area 31. As shown in the literature, commitment, motivation and buy‐in of providers generated acceptance of the club model 15. However, growing numbers of clubs do not automatically represent high acceptance among all club participants. Further research on acceptance among providers, non‐club users and club members would be useful to understand the experience of all participants.

In line with our results, reports identified resources and finances as a jeopardizing factor and others suggested the creation of additional posts to cope with increased workload 11, 30, 32. MacGregor *et al*. 15 attributed a key role to NGOs in providing staff and additional resources. MSF reports called for an adequate drug supply management based on procurement and good pharmacy management as well as policy changes in ART refills 31, 32.

### 4.2 Design and implementation processes

Overall, the *implementation* process of ACs across the Cape Metro District provided a way for clubs to become sustainable. Firstly, the effectiveness of ACs favours sustainability 38. Similarly, other DC models like CAGs and fast track systems succeeded in accomplishing targets such as time‐saving, decongestion and improved clinical outcomes 39, 40, 41. Alongside ACs, patient‐driven CAGs consist of a variable number of stable HIV patients who themselves facilitate a quick symptom check‐up, peer‐based adherence support and community ART distribution 42. For the latter, a group representative visits periodically the clinic to report about the health status of the CAG members and to collect medicines 42, 43. Secondly, the steering committee undertook a crucial role to drive the process forward. The collaborative relationship among stakeholders is a strategy to maximize the success 44. Participation of the committee in negotiation processes led to shared decision‐making which positively influences the clubs’ sustainability 38. The literature recognized the significance of so‐called programme champions to generate sustainability since leading roles help both with the initial planning and the continuity of a programme 45, 46. Conversely, the fading role of the steering committee might be a hurdle for sustaining and expanding clubs. Thirdly, the club implementation was strengthened by mentoring and learning sessions which is acknowledged as one of the enabling factors for sustainability of health interventions 47.

### 4.3 Organizational capacity

The *programme's flexibility* is embedded in the DC approach. The current framework positions the patient at the centre surrounded by four components tailored to specific needs of different patient groups 48. According to Duncombe *et al*. 48 these components include: the location of service delivery, the quality of the healthcare service provider, the frequency of health visits and the type of service delivery. The results of the review presented several examples of adjustments made to cater to the patient's needs. So far, vulnerable patients at risk of loss to follow‐up, unstable patients and patients with co‐infections, had mostly been excluded based on the eligibility criteria 41. Increased efforts have gone into accommodating these groups in clubs 49. Special clubs for adolescents and young people, men and pregnant women, have been introduced 49, 50. While a programme's flexibility is important for sustainable outcomes 51, adaptability to patient's needs is vital for resilient systems 52. This is described as capacity to respond effectively to emerging crises and continuous delivery of high quality service on a daily basis 52, 53. Awareness of threats to this system requires an effective information and surveillance system. In the case of clubs, it means the follow‐up of patient attendance, including, excluded or lost patients and their health outcomes. In addition, the integration of different roles supports health system resilience 52 and therefore information and action should be standardized amongst various club roles. Resilience is associated with learning and transformation to improve 53 and evolve to the needs of the ACs beneficiaries and bring new benefits to the club members, in particular their motivation to remain as members.

A major challenge for clubs is access to resources and finances, especially with declining donor funding for HIV programmes which has negatively impacted the NGO sector in SA 54. Advantageously for SA, most of the HIV efforts are largely financed by the national government and so it is not wholly dependent on donors for funding 55. However, international assistance is still needed to support the largest ART treatment programme as the SA government has limited capacity to mobilize greater resources, due to various factors; low economic growth, the volatile currency and high government's debts 2. The greatest external donors for HIV and TB programmes are the Global Fund and the United States Government 56. As SA reaches a middle‐income country status, the United States President's Emergency Plan for AIDS Relief plans to phase out funding 57 to transfer all programmes to the SA government and to serve solely as a technical support partner 58. Inevitably, this transition process entails risks of replacement strategies being inadequate and create financial gaps between required and available funding 58, 59. Declining donor funding may also impact on the role of NGOs as donor funds contributed to a flexible support for NGOs 55. Currently, SA is moving towards a universal health coverage through implementing a national health insurance by 2030 60. Yet, the lack of published evidence around cost‐effectiveness of the clubs limits the evaluation of clubs’ contribution to universal health coverage.

The apparent staffing bottleneck indicated an increased threat to clubs’ sustainability. The challenge to establish sufficient staff capacity is dependent on various organizations. Frequently, lay counsellors are employed by different organizations leading to poor cohesion amongst teams and sustainability 61. Similar findings were shown in other settings where human, financial and logistical resources are mostly provided by NGOs 41, 62.

Effectively used resources, such as a reliable drug supply, strengthen the sustainability of clubs 63. According to a MSF report, weakness in the drug supply chain management was discovered when 22% of medicine shortfall was in ARV/TB medicine 64. The medicine supply chain in SA is decentralized and each province has its own medicine depot 64. Each province manages distribution, ordering and predicting medication demand 64. As a result, there is a great gap in supply management among the provinces in SA. The Western Cape Province encountered the lowest stock supply issues by far, whereas provinces like Mpumalanga, Limpopo, North West, Free State and Eastern Cape dealt with the majority of medicines shortage 64. Noticeably, rural communities are affected most by shortages in their supply chain and inadequate response management 65. There is a need for national policies to improve monitoring and supply chain management 65. The Western Cape government pushed forward the privatized CDU system with the aim to improve the medicine access and assuring consistent supply 66.

### 4.4 Community embeddedness

*Community embeddedness* is one of the key arguments for the clubs’ sustainability and the cornerstone of DC models. The beneficial outcomes of peer support and active involvement in treatment were confirmed in other settings 41, 62, 67, 68, 69. Patients turn into experts in their treatment, from social support during group meetings and through sharing and combining their knowledge and experience 70. As a result, group meetings have a great potential to generate awareness and empowerment and impact quality of treatment 71. The affiliation to others with similar problems reduces the feeling of anxiety and uniqueness 72, 73. Receiving information and coping strategies as well as social support were discovered as important motivational factors to join HIV support groups 74. Peer support programmes like ACs with emphasis on self‐management and empowerment of patients are also attractive models for the chronic disease management in high‐ and low‐income countries 71, 75. Beyond active treatment involvement, community participation in planning and implementing leads to community ownership and favours sustainability 19, 47. This was shown in Mozambique and Haiti where community stakeholder involvement was used to set up CAGs leading to creation of community ownership 67, 76.

### 4.5 Enabling environment

*Political support* was partially present in SA. Yet more political support is needed to sustain clubs especially on guidelines on the operation of CACs, expansion and sustainability of clubs, reliable drug supply and not forgetting policies regulating LHCWs’ role and scope of practice. To combat these issues, strategies were proposed such as the integration of LHCWs, adequate payment and continued training of staff, based on regulatory frameworks 77. In addition, studies called for recognition of LHCWs, the analysis of workload for the lay counsellor (and the inclusion in national human resources for health data) to improve forecasting and planning for staff demand 61, 62. In SA, lay counsellors fall under the category of community healthcare worker where the funding comes both from government and donors 78. Their scope of practice is vague and lacking in definition and standardization 78, 79. This may increase the risk of insufficient training and dependence on government priorities 61. Good governance includes the development of guidelines and policies to help expand clubs and setting‐up specialist ones, which all favour sustainability 63, 80.

### 4.6 Context

*Context*‐related issues such as the political environment, the economic situation of SA, geographical factors, educational level of patients and cultural norms could favour or threaten the sustainability of ACs. The review sheds little light on these issues and their impact on clubs. The unequal socio‐economic circumstances across SA may influence the transferability of clubs to other provinces and its sustainability. According to the current investigation on poverty in SA, the country faces severe challenges of high unemployment rate, poor quality of education, inadequate infrastructure and a stressed public health system struggling to meet the standards of quality 81. Above all, the socio‐economic factors are unequally distributed within the country resulting in some provinces encountering a stronger burden (such as the Eastern Cape and Limpopo) than others 81. In this context, the province of Western Cape and Gauteng have the lowest levels of poverty 81. However, there is a strong spatial dimension of poverty inside each province where poverty is concentrated in former homelands 82. These inequalities are also mirrored in the availability of qualified healthcare workers 78. Publications on ACs focus on the Cape Town area and are speculative as to how clubs would operate in lower socio‐economic settings. Given the evidence, ACs seem more eligible for urban or peri‐urban settings 28, 29, whereas CAGs are successfully implemented in rural areas like in Mozambique, Malawi and Lesotho, with improved adherence to ART and reduced loss to follow‐up 62, 69, 83.

### 4.7 Limitations

The study has its strengths and limitations. The main strength of the review is the exhaustive search of peer‐reviewed publications based on multiple search strategies including databases, snowball sampling and websites to identify relevant literature. This review followed a systematic process to provide an overview of the current state of evidence for ACs. Studies from different authors and different programmes were retrieved supporting the validation of findings. Despite these strengths, there are some limitations.

The methodological approach of a scoping literature review focusses on the provision of breadth rather than depth of information. A quality assessment of the included studies was not conducted in the review. The review provided a descriptive overview of the available literature for ACs but did not allow a quantitative estimation of the sustainability of clubs.

The process of data selection and the identification of framework components was conducted by a single person which may have led to an incomplete core review and a potential research bias.

There exist limitations of the data reported. Firstly, the review may have not identified all records for ACs in the published literature despite the effort to be comprehensive. This is due to the screening process where only the first 100 records were used which may have led to selection bias. However, the search strategy did identify the first publication on the AC model and the same citations were repeatedly retrieved by different search strategies. Secondly, publication bias exists as positive outcomes are more likely to be published. Thirdly, the lack of qualitative studies investigating the experience of club members, non‐members, staff and other stakeholders limited the evaluation of their perception on clubs and their daily operation. The absence of qualitatively based approaches was also confirmed in a recent review focusing on effectiveness of group‐based care models in sub‐Saharan Africa 14. A final limitation is associated with the generalization of results since the concept of ACs is context‐related and thus cannot be transferred easily from one setting to another without any adjustment.

## 5 Conclusions

The review identified strong enablers of sustainability of the AC model in the Western Cape of which the strongest referred to the programme's flexibility, acceptance among providers and users, the successful implementation approach and community participation. Also, the growing political acceptance of ACs as a strategy to overcome overburdened healthcare systems and to improve clinical outcomes, generates an environment for sustainable development in the future.

However, several weaknesses were recognized with the potential to undermine the clubs’ sustainability. The major obstacles are attributed to (1) the demand for human resources including defined roles and standardized training, (2) high dependence on NGOs and NPOs and (3) funding of future clubs while current donors are phasing out. Possible pitfalls are poor relationships between clubs and facilities, lack of policies for LHCWs, inconsistent drug supply and the declining leadership role of the steering committee.

To meet the national and international targets to fight HIV, a national scale‐up plan for ACs across the country led by the government may be a first step to achieve long‐term sustainability.

Taking into account the socio‐economic differences between and within provinces, other community‐based models other than ACs may present a possible solution for patient‐centred ART delivery.

Only a limited number of peer‐reviewed qualitative studies were available that may have supported the analysis of sustainability. In addition, the retrieved literature only provided evidence on the sustainability of ACs in the Western Cape. In the light of these challenges, further research is suggested to help to understand the experience of patients (peer support and participation) and staff within and outside of clubs and to aid development of effective policies for the clubs. More evidence is required on the transferability of the clubs to other settings, the future role of the steering committee and sources of finances.

## Competing interests

The authors have no competing interests to declare.

## Authors’ contributions

KF designed the study, wrote the first manuscript and conducted the literature review. TD, RvdP and BvdB critically reviewed and commented on drafts of the manuscript to improve the manuscript. TD and BvdB gave advise on the structure of the paper. All authors have read approved the final version.

## Supporting information


**Appendix S1:** Data extraction sheet.
